# 68722 Role of ER calcium in beta cell senescence and diabetes pathophysiology

**DOI:** 10.1017/cts.2021.446

**Published:** 2021-03-30

**Authors:** Staci A. Weaver, Tatsuyoshi Kono, Farooq Syed, Robert Bone, Carmella Evans-Molina

**Affiliations:** Indiana University School of Medicine

## Abstract

ABSTRACT IMPACT: The proposed study has the potential to inform new paradigms of type 1 diabetes prevention and therapy with the overall goal of improving β cell health during autoimmunity. OBJECTIVES/GOALS: Type 1 diabetes (T1D) results from immune-mediated destruction of pancreatic βcells. Recent data suggest that activation of senescence and acquisition of a senescence associated secretory phenotype (SASP) by βcells may contribute to T1D pathogenesis. However, the molecular mechanisms responsible for this phenotype are not well understood. METHODS/STUDY POPULATION: We hypothesize that loss of endoplasmic reticulum (ER) Ca2+ induces βcell senescence, SASP as well as mitochondrial dysfunction which drive T1D development. The current study utilizes SERCA2 KO INS-1 βcells (S2KO) exhibiting loss of ER Ca2+ and a SERCA2 haploinsufficient mice on a non-obese diabetic background (NOD-S2+/-) to test the role of ER Ca2+ loss during T1D development. Senescence associated βgalactosidase staining (SA-βgal), expression of senescence markers (RT-qPCR), mitochondrial function (Seahorse, TMRM) and mitochondrial copy number (qPCR) were all measured in S2KO versus WT βcells and are currently being measured in the NOD-S2+/- mouse model at 6, 8, 12, 14, and 16wks of age. RESULTS/ANTICIPATED RESULTS: RT-qPCR assays detecting senescence markers cdkn1a and cdkn2a and mitochondrial specific genes cox1 and nd1 were developed and validated in both INS-1 βcells and mouse islets. Mitochondrial function assay (Seahorse) was optimized for use in INS-1 βcells and is currently under development for use in intact mouse islets. S2KO βcells displayed increased SA- βgal staining as well as increased mitochondrial coupling efficiency (p=0.0146) and baseline mitochondrial copy number (p=0.0053) compared to WT βcells, suggesting a senescence phenotype and altered mitochondrial function. NOD-S2+/- mice exhibited increased expression of the senescence marker cdkn2a in the islet at 12wks (p=0.0117) compared to control mice, whereas cdkn1a remained unchanged across all timepoints tested. DISCUSSION/SIGNIFICANCE OF FINDINGS: Our results suggest that loss of SERCA2 and reduced ER Ca2+ alter βcell mitochondrial function and are associated with features of senescence. Future studies will test whether SERCA2 activation and/or senolytic/senomorphic drugs are able to prevent or delay diabetes onset in NOD-S2+/- mice.

